# EPIphany—A Platform for Analysis and Visualization of Peptide Immunoarray Data

**DOI:** 10.3389/fbinf.2021.694324

**Published:** 2021-07-07

**Authors:** Zoe Parker Cates, Antonio Facciuolo, Daniel Hogan, Philip J. Griebel, Scott Napper, Anthony J. Kusalik

**Affiliations:** ^1^ Department of Computer Science, University of Saskatchewan, Saskatoon, SK, Canada; ^2^ Vaccine and Infectious Disease Organization (VIDO), University of Saskatchewan, Saskatoon, SK, Canada; ^3^ School of Public Health, University of Saskatchewan, Saskatoon, SK, Canada; ^4^ Department of Biochemistry, Microbiology and Immunology, University of Saskatchewan, Saskatoon, SK, Canada

**Keywords:** immunoarray, immunosignature, data normalization, epitope analysis, antibodies, data visualization, web service

## Abstract

Antibodies are critical effector molecules of the humoral immune system. Upon infection or vaccination, populations of antibodies are generated which bind to various regions of the invading pathogen or exogenous agent. Defining the reactivity and breadth of this antibody response provides an understanding of the antigenic determinants and enables the rational development and assessment of vaccine candidates. High-resolution analysis of these populations typically requires advanced techniques such as B cell receptor repertoire sequencing, mass spectrometry of isolated immunoglobulins, or phage display libraries that are dependent upon equipment and expertise which are prohibitive for many labs. High-density peptide microarrays representing diverse populations of putative linear epitopes (immunoarrays) are an effective alternative for high-throughput examination of antibody reactivity and diversity. While a promising technology, widespread adoption of immunoarrays has been limited by the need for, and relative absence of, user-friendly tools for consideration and visualization of the emerging data. To address this limitation, we developed EPIphany, a software platform with a simple web-based user interface, aimed at biological users, that provides access to important analysis parameters, data normalization options, and a variety of unique data visualization options. This platform provides researchers the greatest opportunity to extract biologically meaningful information from the immunoarray data, thereby facilitating the discovery and development of novel immuno-therapeutics.

## Introduction

Antibodies are critical effector molecules of humoral immunity. Through their ability to recognize and bind specific targets (epitopes) these proteins serve as a critical line of defence by neutralizing potential threats while activating higher-level immune responses. Through infection or vaccination, there is virtually limitless potential to generate antibodies with the capacity to uniquely recognize different protein sequences and structures, and to form long-lived immune memory. With that, the antibody population present within mammals offers valuable insight into their past, present, and future health. This complex and diverse population of antibodies reflects the immunological challenges that the organism has encountered, is currently prioritizing, and is prepared to face. Detailed accounting of the reactivities represented within this population can identify biomarkers with utility for diagnostic applications. For example, shifts in the reactivities of the population in response to a stimulus, like infection, inform the immunological nuances of the host-pathogen interaction, information that can be applied to guide rationale design of vaccines as well as disease diagnosis and prognosis.

There are several features of antibodies that are well suited for high throughput omic investigations. These vast, complex, and dynamic antibody populations are easily sampled at several minimally invasive anatomical sites (e.g., blood, sputum, feces, colostrum/milk, saliva, tears, mucus from nose, throat, or genital area). In terms of the magnitude and complexity of the antibody population, the immunoglobulin G (IgG) antibody population has an estimated capability for recognition of greater than 10^15^ molecular targets (Rees, 2020). This provides the capacity for highly nuanced immunological responses as well as highly individualized immunological profiles, important features for biomarker discovery and application. These antibody populations are also highly responsive; antibody-secreting cells can generate 10^11^ copies of a specific antibody within a week (Sykes et al., 2013) providing a natural amplification of signal that benefits efforts to characterize changes within the population. Finally, structural characteristics of antibodies are ideally suited for high-throughput investigation in that they consist of unique complementary-determining regions within the Fab arms at the amino-terminal end of the molecule that enable specific recognition of targets, as well as a structurally conserved Fc region at the C-terminal end that facilitates detection of the entire population, or a specific isotype, using a common detection method.

**FIGURE 1:**
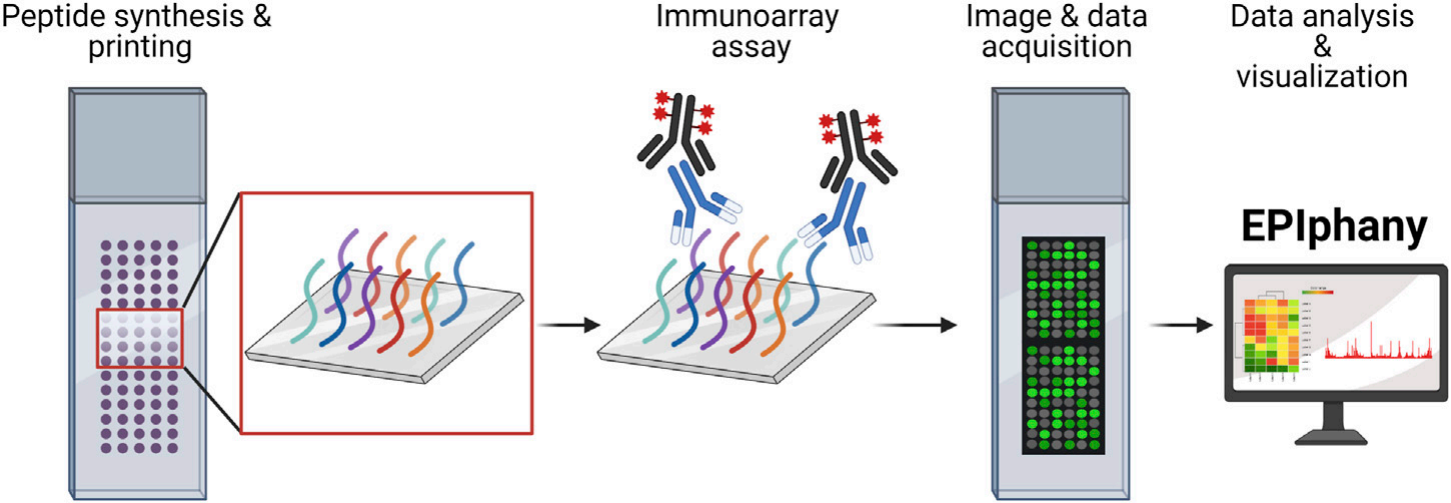
Overview of Design and Implementation of Immunoarrays. Custom peptide synthesis and printing onto a solid-phase matrix, with the capacity of over 6,000 unique peptides, is available from several commercial manufacturers. Immunoarray assays are like conventional ELISAs involving incubation with a primary sample (serum, plasma, or purified antibodies) and detection using a fluorescent-conjugated reporter molecule or antibody. Imaging of peptide arrays requires the use of a microarray scanner and companion software to translate images into numerical data as GPR or CSV files. The GPR or CSV files are uploaded into EPIphany to process the array data, perform statistical analyses, examine data characteristics, and generate publication-ready visualizations. The schematic was generated using BioRender.

Global characterization of the reactivities present within antibody populations have largely been performed through either phage display, NextGen sequencing, or mass spectrometry (Sykes et al., 2013). While these approaches have demonstrated degrees of success, they are commonly disadvantaged by their requirement for highly specialized and expensive equipment, as well as substantial technical expertise. Immunoarrays are a promising technology for rapid, global surveys of the reactivities represented within a population of antibodies. These arrays measure the reactivity of antibodies toward an array of peptides representing potential antigenic determinants. With these arrays, short peptides, typically ranging from 14 to 26 amino acids in length, are presented on a scale of thousands of unique sequences, each localized to unique coordinate on the surface. The immunoarray is fundamentally related to an enzyme-linked immunosorbent assay (ELISA) in that peptides affixed to a solid phase are reacted with serum, plasma, or purified antibodies and antigen-antibody complexes are detected using reporter-conjugated secondary antibodies (Figure 1). The higher capacity and superior assay sensitivity of immunoarrays facilitates more effective high throughput screening when compared to ELISAs.

The sequences of the peptides on an immunoarray can be strategically selected to represent potential antigens within the proteome of a microbe of interest. For example, an array representing the entirety of the proteome of SARS-CoV-2, as well as four other pathogenic human coronaviruses, was recently reported (Holenya et al., 2021). In the case of arrays for autoimmune disorders, sequences from specific self-proteins are represented (Hecker et al., 2016). In either scenario, overlapping peptides can be constructed to enable mapping of reactivities to specific epitopes. Alternatively, peptide microarrays representing random sequences enable unbiassed survey of the reactivities within the antibody population yielding novel immunosignatures (defined as a pattern of reactivities generated by circulating antibodies) that accurately diagnose certain disease states despite the primary sequences of those peptides having no apparent relationship to protein antigens involved in the disease (Chapoval et al., 2017).

Immunoarrays have been applied in mapping antigenic determinants (epitopes) of proteins, antibody signature profiling, and discovery of disease biomarkers (including of autoimmune disorders) (Restrepo et al., 2013; Hecker et al., 2016) as well as responses to vaccines (Legutki et al., 2010; Legutki and Johnston, 2013). Within the realm of host-pathogen interactions, immunoarrays have been applied as diagnostic tools to determine if exposure to a particular pathogen has occurred as well as to understand specifics of host antibody response to a particular pathogen (Holenya et al., 2021). Immunoarrays can guide the development of vaccines through identification and translation of neutralizing epitopes that occur in the context of natural infection into vaccines, as well as evaluation of the ability of candidate vaccines to induce antibodies with reactivity to these priority targets. Lastly, immunoarrays have generated novel observations that have led to a shift in our fundamental understandings of the antibody-antigen interaction (Sykes et al., 2013).

The prioritization of linear, or continuous, epitopes by the immunoarrays also facilitates the rapid translation of these targets into existing platforms for creation of peptide-based vaccines (Malonis et al., 2020). Many antibodies generated in response to infection recognize and bind to their cognate antigen in its folded or conformational state (i.e., conformational or discontinuous epitope). While immunoarrays are limited to linear peptide sequences lacking higher-order structures, there are ample examples of protective antibody responses which are not dependent upon conformational epitopes. Recent computational advancements have even pioneered novel strategies to design linear peptide sequences that mimic conformational epitopes which might expand the utility of immunoarrays into the realm of discontinuous epitopes (Mayrose et al., 2007). In addition, peptides that mimic epitopes despite being dissimilar in primary sequence (mimotopes) have been exploited for their remarkable ability to bind antigen-specific antibodies with the same affinity as the cognate epitope and can even mimic the epitope by eliciting similar humoral immune responses (Riemer et al., 2004). Thus, peptides have much more potential and value beyond their primary sequence, despite lacking higher-order structures, that remains largely unexplored and could contribute to a range of novel immunotherapeutic applications.

The use of immunoarrays to characterize antibody populations is an underutilized experimental approach with considerable opportunities for further refinement and optimization (Szymczak et al., 2018). Extracting meaningful biological information from the immunoarray is not a trivial exercise and it is important not to underestimate the volume, and technical and biological complexity of the emerging data. Due to similarities in format, immunoarrays present many of the same challenges as DNA microarrays (e.g., multiple hypothesis correction and normalization). However, they also present several unique difficulties that warrant special software (e.g., the lack of calibration probes). Several public web applications analyze user-submitted immunoarray data to identify binding motifs and profiles, but do not compare binding signatures across cohorts, including ArrayPitope (Hansen et al., 2017) and SVM-PEPARRAY (Chen et al., 2009). Additionally, tools such as rapMad (Renard et al., 2011), pepStat (Imholte et al., 2016), and pepBayes (Imholte and Gottardo, 2016) are available to compare binding signatures from different cohorts, but only exist as R packages. Collectively these resources face several critical limitations: requirement of computational biology expertise outside the realms of many biological researchers, the absence of a web-based interface, the need to be run locally by the user, insufficient resources for statistical interpretation, an absence of data normalization options, and a lack of tools for visualization of the results.

To make immunoarray technology more easily accessible to researchers of all backgrounds, we have developed a program called EPIphany (“EPItope arrays Pose Hard ANalYsis problems”) which provides to the research community a web service featuring an intuitive user interface to analyze immunoarray data. EPIphany requires no registration or software installation, provides a simple user interface online with access to important analysis parameters and data normalization options, and produces unique data visualizations. This provides researchers the greatest opportunities to extract biologically meaningful information from immunoarray data to facilitate the discovery and development of novel immuno-therapeutics.

## Materials and Methods

### Data Input

EPIphany expects spot intensity data in one of two formats: GenePix results (GPR) or CSV (comma-separated values). The GPR format is ideal if the data is directly from GenePix(R) Pro Microarray Analysis Software. For all other cases, the data should be preprocessed to conform to the CSV layout described below. Files from each cohort (“treatment” vs. “control”) are uploaded in separate batches so treatment and control metadata is not explicitly required within the entered files.

The uploaded CSV files must be text files containing the following four columns in order: 1) an ID (identification) column uniquely identifying each spot, 2) a peptide sequence column describing the sequence of the peptide contained at each spot, and 3) foreground and 4) background columns quantifying the measured intensity of each spot. Regardless of the format, spot intensity data from each array (sample) must be uploaded in an individual file.

EPIphany allows the user to select the nature of calculated spot (peptide) intensity values. This can be foreground intensity, background-corrected foreground intensity, or background-scaled foreground intensity. For background-corrected foreground intensity, the background intensity value for a spot (peptide) is subtracted from the given foreground intensity to yield a peptide-specific intensity value. This technique attempts to account for localized, systematic variations in spot intensity. Background scaling is comparable to background subtraction except that prior to the difference calculation, both intensities are first divided by the ratio between the local background intensity and the median background intensity of the entire array (Eq. 1).

fs=fl(blbm)−bl(blbm)=flbmbl−bm (1)

*f*_*s*_ is the scaled final foreground intensity for the target peptide (*f*). *f*_*l*_ and *b*_*l*_ represent the original local foreground and background intensities for *f* and *b*_*m*_ is the median background intensity of the entire array.

This ratio is an additional factor accounting for location bias by considering the relationship between the local background (for the peptide under consideration) and the background across the entire array.

### Data Normalization

The distribution of peptide reactivity across arrays may differ significantly due to uncontrolled experimental variables, which is an inherent problem with microarrays. Two assumptions can be invoked, which lead to two possible normalization methods. The first is that the distribution of peptide reactivity in the different arrays is approximately the same. The natural normalization technique under this assumption is quantile normalization (Schmid et al., 2010). The second assumption is that peptide reactivity is an affine transformation of the ground truth. The variance stabilizing normalization (VSN) (Huber et al., 2002) furnishes the appropriate technique under this assumption, while also correcting heteroscedasticity; however, a precondition for accurate fitting of the VSN transformation is that many peptides are not differentially reactive. EPIphany also provides the option of no normalization.

### Synoptic Analysis

EPIphany combines the uploaded datasets and performs the selected normalization using the specified type of intensity values (foreground, background-corrected foreground, or background-scaled foreground). It also performs a Mann-Whitney U-test for each spot (peptide) to determine whether the distribution of peptide intensities in the treatment datasets is the same as the distribution of peptide intensities in the control datasets. The null hypothesis is that, for a given peptide, the two distributions are the same while the alternate hypothesis is that they are different. A *p*-value is calculated using the U-test. Since multiple hypotheses are being tested using the same datasets, an adjustment for this is provided using a Benjamini-Hochberg correction. The user can select a tolerated false discovery rate (FDR) of 0.01, 0.05, 0.10, or 0.25, and adjusted *p*-values are produced. Two of these thresholds are higher than “typical” because, in this application, false negatives are more problematic than false positives. This data manipulation is a screening exercise to find candidates rather than a verification exercise to confirm candidates and there are subsequent stages of analysis with which to eliminate false positives. With that, more tolerant *p*-value thresholds are useful. The results of this initial synoptic analysis can be downloaded by the user for local offline analysis using a regular spreadsheet program. The results can also be utilized for subsequent, targeted visualization. Synoptic analysis also provides a number of plotting options (e.g., boxplot, clustering plot, mean vs. variance plot, and mean vs. median plot) to both visualize and analyze characteristics of the entire dataset. In addition, the initial analysis available at this stage can generate heatmaps, swarm plots, and dendrograms focusing on specific subsets of the data formed based on the degree of differential reactivity of peptides, statistical significance, or both.

### Targeted Visualization

EPIphany can produce additional visualizations that require information beyond that in the initial GPR or CSV files with spot intensities that are restricted to a small subset of peptides (to yield a legible plot). This supplementary information includes identity of the pathogen involved in the experiment, the protein from which peptides are derived, and the start and end positions of a peptide within its source protein. In addition, the subset of peptides to consider is explicitly specified by the user rather than being automatically selected as in the synoptic visualizations. To obtain these additional visualizations then, the user must upload a targeting file with this supplementary information. Upon upload the user can then select from a line graph, an epitope map, as well as an enhanced heat map and strip plot. For the first three types of plots, the targeting file should specify a contiguous sequence of positions within a single protein. For a strip plot, on the other hand, the targeting file can specify a collection of arbitrary peptides (i.e., not necessarily in contiguous positions) from a given protein.

The characteristics of the four types of visualizations are as follows. The heatmap is similar to that produced within the synoptic analysis. However, since position information is now provided to EPIphany in the targeting file, the y-axis of the heatmap can correspond to peptide position within the specified source protein rather than a perhaps arbitrary spot ID.

The line graph uses three panels to show the trend in control and treatment cohort intensities for the peptides specified in the uploaded targeting file. In the first two panels, a band shows the range of intensity values for the specified peptide positions. The center of the band is shown in white and indicates the mean value, while the edges of the band are ±1 standard deviation from the mean. The third panel shows the effect size; i.e., the treatment mean less the control mean.

The strip plot not only shows the distribution of sample values for each specified peptide color-coded to indicate whether it is from a control or treatment sample, but also maintains a correspondence between the sources for the data points. That is, the dot in position n (from the right or left) within each strip is always from the same sample (dataset).

Lastly, the epitope map shows the location of each peptide on its source protein, with the representation of each peptide color-coded according to its effect size (treatment mean less control mean).

### Peptide Microarrays and Biological Samples

Peptide microarray data was provided from an independent study (Facciuolo et al., manuscript in preparation). GPR files from that study were used to demonstrate the utility and functionality of EPIphany. Thus, only a subset of data from that analysis is reported and shown in the current study. Briefly, RepliTope™ Antigen Collection Pan-Coronavirus (Product Code: RT-HD-CoV2) microarrays were purchased from JPT Peptide Technologies (Berlin, Germany). Each array consists of 4,416 peptides covering the full proteome of SARS-CoV-2, and spike glycoprotein, nucleoprotein, envelope small membrane protein, and membrane protein of SARS-CoV, MERS-CoV, and human coronaviruses HCoV-229E and HCoV-OC43. Each protein is represented by 15-mer peptides with 11 amino acid overlap and printed in triplicate. Each individual array was incubated with serum from humans diagnosed with COVID-19 5 months prior (*n* = 22) or from humans with no previous exposure to COVID-19 (*n* = 20).

### Immunoarray Assay

All incubation steps were performed at room temperature on a rotating shaker. Peptide microarrays were blocked in Tris-buffered saline (TBS), pH 7.2 supplemented with 0.05% v/v Tween-20 (TBS-T) and 3% w/v bovine serum albumin fraction V (BSA; diluent) for 30 min. Serum was diluted 1:100 in diluent and incubated for 2 h. Each array was washed with 5 exchanges of TBS-T, and once with sterile deionized distilled water. Primary antibody was detected using Alexa Fluor 647 conjugated secondary antibody diluted to 1 μg/ml in diluent and incubated for 45 min in the dark. Washes were carried out as described above, and slides dried by centrifugation for 5 min at 800 × g.

### Immunoarray Image Acquisition

Peptide arrays were imaged using a GenePix Professional 4200A microarray scanner (MDS Analytical Technologies, Toronto, ON, Canada) equipped with a 635 nm laser and fluorescence captured using a 655–695 nm filter. Images were scanned at 10 µm resolution and data acquired using GenePix software (version 6.0).

## Results

**FIGURE 2:**
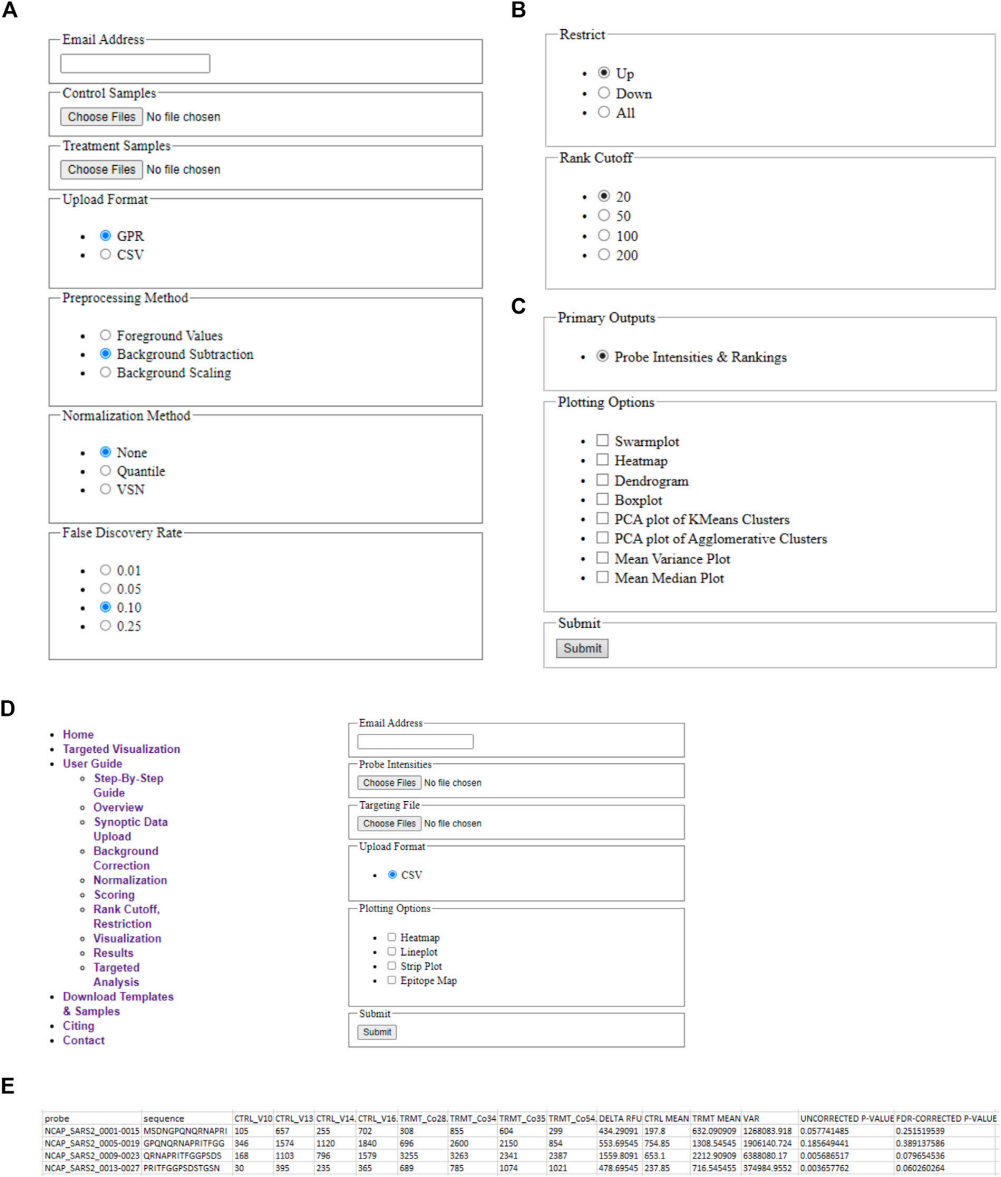
EPIphany Workflow and User-Interface. **(A)** EPIphany provides an easy-to-use interface for uploading data, selecting data preparation methods, **(B)** performing primary analyses to determine the most differentially reactive peptides, and **(C)** generating plots to examine data characteristics (PCA plot, M/V plot, etc.). **(D)** Additionally, EPIphany offers follow-on, targeted data visualizations using user-specified data subsets. All the features can be accessed and executed without the need for any higher-level computational expertise. **(E)** Sample table displaying the first five rows of the CSV file that is provided to the user after synoptic analysis of uploaded datasets.

A schematic illustrating the immunoarray pipeline from peptide design to implementation is shown (Figure 1). EPIphany offers a simple user-interface (Figure 2) and workflow (Supplementary Figure 1) in which primary, or synoptic, analyses and deeper, targeted analysis can be performed. Synoptic analysis includes steps such as data processing and generating informative graphs (e.g., cluster plots, mean-variance plots) to describe data characteristics as well as providing part of the input for the targeted analysis (Figures 2A–D). Supplementary Table 1 details the Python library functions used for the major analyses and visualizations provided by EPIphany, and the important parameters in those function calls; parameters not mentioned are left at their default values. On the EPIphany server, a collection of files is produced with the results of the synoptic analysis. The most important of these is a CSV file with columns that include the spot ID and peptide sequence from the input GPR or CSV files, plus columns for the potentially adjusted and normalized intensity value from each sample, mean for control and treatment samples, variance for control and treatment samples, “delta RFU” (effect size, in this case the treatment mean less the control mean), raw *p*-value (from the Mann-Whitney *U*-test), and adjusted *p*-value (after FDR correction) (Figure 2E). If visualizations are selected by the user, both synoptic and targeted analyses can result in graphics files being produced. All results files are returned to the user by email.

### Synoptic Visualizations

**FIGURE 3:**
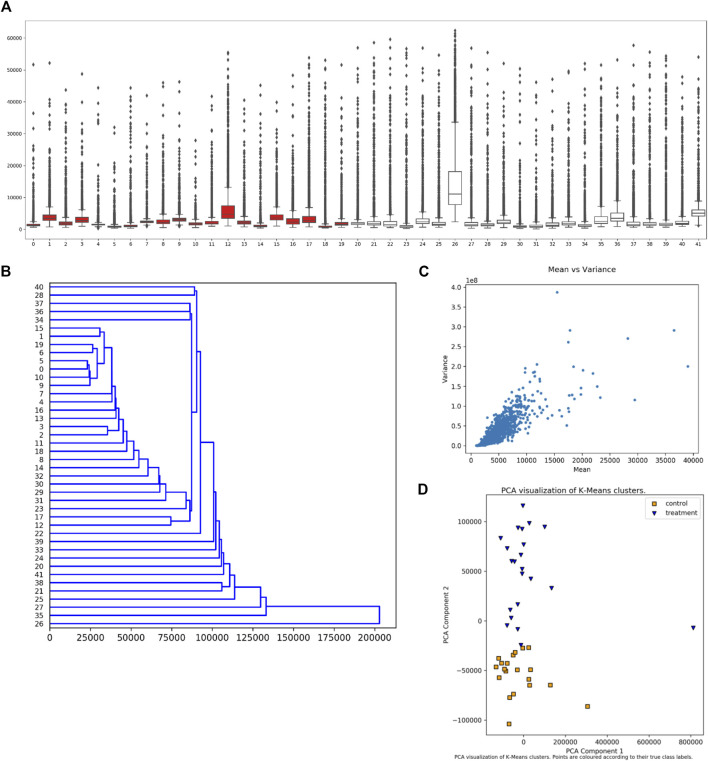
Synoptic Visualizations Available in EPIphany. EPIphany provides a number of visualizations that are available after its initial analysis. These include: **(A)** boxplots or box-and-whisker plots, which show the distribution of all peptide values for each sample (dataset); **(B)** dendrograms, which show hierarchical clustering of samples (input datasets) based on response intensity across a subset of peptides; **(C)** mean vs. variance plots, through which a user can check for heteroscedasticity; and **(D)** PCA (principal component analysis) plots of k-means clustering of control and treatment samples. The samples under consideration label the *x*-axis in the boxplot **(A)** and y-axis in the dendrogram **(B)**. Intensity is shown on the y-axis in the boxplot **(A)** and x-axis in the mean-variance plot **(C)**. In the boxplot **(A)**, the rectangle indicates the extent of the first and third quartiles, with the line crossing the rectangle indicating the median. Lines extending beyond the rectangle (“whiskers”) indicate observations prior to the first quartile and after the third quartile. Dots beyond the whiskers indicate outliers. For the k-means clustering **(D)**, k is set to 2 reflecting the situation that the samples come from two cohorts, treatment and control. The clustering is performed prior to the PCA.

The user can select from several visualization (plotting) options without requiring further data input. Examples of these plots are given in Figure 3. The plots include a boxplot (Figure 3A), a dendrogram (Figure 3B), mean vs. variance plots (Figure 3C), and two types of clustering plots (Figure 3D), in addition to swarm or strip plots, heat maps, and mean vs. median plots. For dendrograms and clustering plots, for a given sample (dataset), the intensity values for each peptide (suppose N of them) are interpreted as the coordinates of an N-dimensional point (in an N-dimensional space) representing that sample.

If datasets are very large, generating a heat map or swarm plot from the data can be computationally challenging and the resultant image can have poor resolution of detail. The user must therefore select a subset of results to visualize the data using these plots. Via the “Rank Cutoff” setting, the user must restrict the subset to 20, 50, or 100 peptides. In addition, the subset can involve only those peptides where intensity increased or decreased in treatment over control, or any peptide irrespective of direction of intensity change. The latter is controlled by the parameter headed by “Restrict”. Suppose 50 peptides and a direction of “Down” are specified. Swarm plots and heat maps are then produced using the peptides with the 50 lowest corrected *p*-values; the peptides having the 50 least effect sizes (equivalently, the 50 peptides where control mean less treatment mean is greatest); and the 50 peptides with the least effect size and that have corrected *p*-values less than the FDR threshold.

The boxplot (Figure 3A) is created using the uploaded datasets, and all peptides within each dataset. Control samples are shown in red, and treatment samples are portrayed in white. A boxplot allows a user to quickly see the overall characteristics of their data, and, if any sample (dataset) has a distinctly different distribution of values from other samples in the same cohort.

The dendrogram (Figure 3B) shows hierarchical clustering for each sample (dataset), irrespective of whether it is in the treatment or control cohorts. The peptides used to represent each sample (dataset) shown in the dendrogram are those that satisfy the subset restriction as described earlier. For example, if 20 peptides and a direction of “Up” are specified, then the values for the peptides with the 20 largest effect sizes and with corrected *p*-values that meet the FDR threshold are used to determine the coordinates (in a 20-dimensional space) for each sample. The hierarchical clustering of samples is then computed using “average” linkage (the UPGMA algorithm) and Euclidean distance, and the result plotted.

Clustering of samples (datasets) is performed using the intensity values for each peptide as coordinates in an N-dimensional space representing that sample, where N is the total number of peptides. Two types of clustering are performed followed by PCA (principal component analysis) to present the clusters. Hierarchical (agglomerative) clustering is performed using Euclidean distance and “average” linkage. Since the samples are from two cohorts, the first two clusters (from the first bifurcation) are then selected for plotting. In addition, k-means clustering is performed with *k* = 2. To portray the clusters, the points in N-dimensional space are subjected to PCA (principal component analysis) and the clusters plotted within the first two axes of the resultant space. The clustering and PCA are performed using all input peptides without applying the previously described subset restrictions. Since it is known which sample values are “control” and “treatment” respectively, plots are shown with and without this coding. An example of the resultant plot is given (Figure 3D). Finally, the “quality” of the clusterings is evaluated using Davies-Bouldin, Silhouette, and Calinski-Harabasz indices, and the values provided in tables.

The final plots that can be selected at this stage are mean vs. median and mean vs. variance plots. One of each type of plot is produced for the control cohort (collection of datasets) and treatment cohort. The plots are produced using all input peptide values; no restrictions or filtering is applied. An example of this plot is given (Figure 3C).

To further support the end-user in data analysis and visualization, EPIphany provides subsequent, targeted data analysis and visualization tools. As with all technologies that generate large-scale datasets, a major hurdle for the utilization of the platform is having the necessary tools to extract meaningful biological data. As such, we have tailored the development and implementation of additional visualization tools in EPIphany that uniquely address the needs in displaying immunoarray data. These visualizations are often only available as programming packages thus requiring computational expertise or paid software. In both cases, this can present a major barrier to the adoption of this technology.

### Targeted Visualizations

**FIGURE 4:**
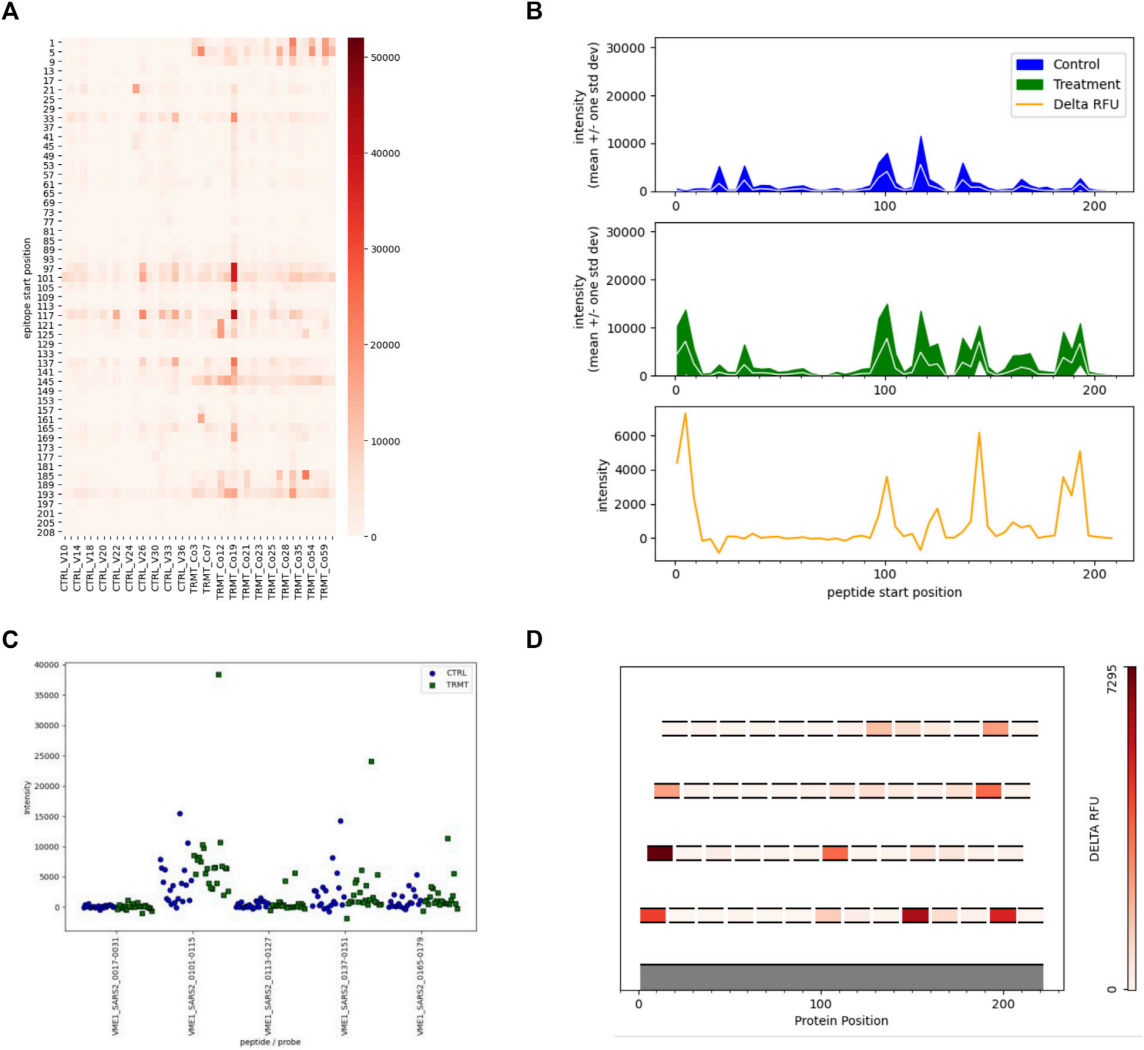
Targeted Analyses and Visualizations Available in EPIphany. EPIphany offers follow-on data visualizations by allowing user-defined tables to be uploaded and choice among the four representative plots. The uploaded tables specify the peptides to be selected for visualization, as well as the pathogen, protein, and peptide position (within that protein) of each peptide. **(A)** Heat maps allow for the visualization of each individual sample reactivity. Peptides listed on the y-axis are arranged N-terminus to C-terminus. **(B)** Line graphs generate an immunosignature by displaying the mean intensity ± 1SD for the control and treatment cohorts, with an additional panel displaying effect size (“delta RFU” or the difference in mean signal intensity between the two cohorts). **(C)** Strip plots show the distribution of spot intensities with more precision than the other types of plots. **(D)** In scenarios where overlapping peptides are used to identify and locate antigenic regions within a polypeptide or protein, epitope maps show the location of individual peptides on an index sequence with effect size of the peptides colour-coded. Epitope maps provide a simple yet effective means to identify the core regions of antibody reactivity.

In this initial version of EPIphany we focused on the development of four unique visualization tools that best display meaningful biological data and generate publication-ready figures (Figure 4) for the end-user. For each of these unique visualizations, the user is instructed to simply upload two CSV files: one with the unmodified results of the synoptic analysis and an additional file (targeting file) containing supplementary information about a subset of data for further analysis (Figure 2D). The targeted heat maps in EPIphany arrange peptides along the y-axis representing their spatial position along the linear protein sequence (Figure 4A). The user specifies a consecutive set of positions to consider in the targeting file. This facilitates either a global, comparative overview of the signal intensity across the full-length protein for each individual sample tested, or a more focused analysis if a subset of the protein is specified. Alternatively, peptide reactivity across a linear protein sequence can be represented using line graphs (Figure 4B) which display a continuous signal intensity line generating a distinct “immunosignature”. In EPIphany, immunosignatures are generated for the mean ± 1 standard deviation for the control samples and experimental samples. Additionally, a third panel generated alongside these line graphs augments the immunosignature by showing the effect size [calculated by subtracting the baseline control mean from the experimental (or treatment) mean] allowing for the quick visual identification of antigenic regions that are differentially reactive between two datasets.

In scenarios where overlapping peptides are employed to map antigenic regions, EPIphany offers the user an epitope mapping visualization tool whereby each peptide is mapped along an index sequence (i.e., full length protein sequence, or contiguous portion thereof) and displayed as a function of its effect size, a color-coding similar to a heat map (Figure 4D). This allows a user to identify the core reactive region among a set of overlapping peptides. Lastly, as the aforementioned tools provide a global visualization of peptide reactivities against the backdrop of a linear protein sequence, EPIphany also provides the user with the option of generating strip plots to facilitate a more detailed perspective of how each sample within the population tested reacts with peptide(s) of interest (Figure 4C). Thus, EPIphany provides a range of visualization tools from global analysis to more detailed perspectives that dually serve to provide the end-user with both publication ready figures and enable a superior visual analysis of peptide reactivity.

## Discussion

Immunoarrays offer tremendous potential to characterize complex populations of antibodies, including specific application to understand host-pathogen interactions, to enable the discovery and application of diagnostic biomarkers as well as for strategic design of vaccines. While the most notable potential contribution of the immunoarrays to vaccine development is within the context of identifying vaccine targets, they can also inform strategies of formulation and delivery, including adjuvant selection, to impact the magnitude, breadth, and specificity of the humoral immune response. Immunoarrays also provide a valuable tool to assess the vaccine-induced reactivities of antibodies to naturally occurring variants of a particular pathogen.

Immunoarrays are yet to reach their full potential, both in terms of utilization and quality of information provided. Ease of use and efficiency of extraction of meaningful biological information associated with large-scale datasets, such as peptide microarrays, are a major barrier to enabling the widespread adoption of this technology. Notably, we identified and addressed a similar deficiency for the use and application of peptide arrays for kinome analysis a decade ago (Li et al., 2012). The software platforms we developed for the design (Trost et al., 2013a; Trost et al., 2016) and interpretation (Li et al., 2012; Trost et al., 2013b) of peptide arrays for kinome analysis have found widespread utilization (Facciuolo et al., 2020). Our goal with EPIphany is to provide a similar resource for any novice to experienced end-user to capture and analyze data emerging from the use of peptide arrays, and in particular offer the user valuable tools for data manipulation and visualization for immunoprofiling. In turn, this will help to accelerate the use of this high-throughput technology for analysis of antibody responses and serve as a catalyst for the next stage of evolution for peptide microarrays.

For data manipulation, EPIphany provides the user with the opportunity to apply three different strategies of data normalization: no normalization, VSN, and quantile. Several papers describe immunoarray results obtained in the absence of data normalization that have been validated through independent techniques, and arguments have been presented against the need for any normalization (Hecker et al., 2012). Other reports have demonstrated added value in applying various normalization methods (i.e., linear model and Bayesian hierarchical modeling) and data pre-processing methods (i.e., correcting for spatial and systematic biases) for discriminating binding signatures between cohorts in a vaccine study (Renard et al., 2011; Imholte et al., 2016; Imholte and Gottardo, 2016). At the present time, we feel it is premature to discount the value and appropriateness of data pre-processing and normalization methods given there is no consensus among the current end-users. It is unclear which, if any, data normalization approaches will be most appropriate for peptide microarray data and the question warrants further study. While each approach has demonstrated utility for various types of omic-associated biological data, the data emerging from different omic approaches have distinct biological and technical characteristics. The motivation behind including the three normalization approaches is to provide opportunity for users to investigate different approaches to determine which is the most effective for extracting meaningful biological information from their immunoarray datasets. The “correctness” of a normalization approach could be evaluated by the ability of the manipulated datasets to cluster based on a particular phenotype (for example, infected individuals vs. non-infected controls) or by more targeted validation of the results of individual epitopes through ELISA. As a consensus emerges of which normalization and data pre-processing approaches are the most appropriate, EPIphany will be modified to reflect that consensus, through addition/elimination of the other normalizations and presenting a specific approach as the default.

For data visualization, EPIphany offers several tools that rapidly allow the user to identify overall trends with the datasets, including different immunoprofiles corresponding to different treatment groups (for example, control vs. infected), as well as different phenotypic outcomes, such as severity of infection. Visualizations of reactivities within specific proteins enable the user to rapidly assess hot spots of reactivity, the extent of cross-reactivity of the same protein from related species, and the nature and specificity of antibody responses emerging from either vaccination or infection. Similar visualization tools are available using R peptStat package (Imholte et al., 2016) in addition to other R add-on packages. However, access to these tools is limited to those with advanced computational backgrounds and places the use of these tools out of reach for many biological end-users. EPIphany was designed with the intent of placing these tools in the hands of biological researchers, and to present “publication ready” figures to facilitate the rapid decimation of results.

This is an ideal time for the development of a user-friendly platform for analysis and presentation of immunoarray data. Firstly, while the application of the technology has been largely limited to a small number of labs, the findings to date highlight the potential of the approach and will inevitably attract new adopters. EPIphany will lower some of the activation barriers associated with incorporating a new technology into a research program and will serve as a platform for discussion to help generate a consensus on standards and protocols for the handling and interpreting of peptide microarray data to ensure accuracy and reproducibility across labs. The COVID-19 pandemic is also likely to spur further interest in high-throughput, low-technology approaches that enable global survey of antibody populations. Notably, in response to the pandemic JPT generated and marketed a customized array representing the entirety of the proteome of SARS-CoV-2 as well as that of other human coronaviruses including SARS-CoV, MERS-CoV, and two human coronaviruses associated with the common cold. Finally, we believe that the technology will soon be challenged with questions of greater biological complexity that will require more sophisticated data analysis tools. In particular, there will be priority to address questions regarding the specificity, cross-reactivity, and cross-protection of antibodies.

A positive consequence of the COVID-19 pandemic is the acceleration of the field of vaccinology. This is most apparent in new mechanics of vaccine technologies, most notably with approval of mRNA vaccines. This is also evident in a shift in the priorities and philosophies of vaccine development. The pandemic has raised appreciation of the importance that the speed at which a vaccine can be developed is measured not only in months spent but also lives lost. It was extremely fortuitous that the Spike protein proved a safe and effective vaccine target, but there is also appreciation that other emerging threats may not offer such readily apparent targets. In those instances, it will be critical to have technologies that can rapidly assess the antibody response associated with a protective response to enable rapid translation to vaccines; peptide immunoarrays hold such potential. There is also appreciation of the importance of the cross-reactivity of the induced antibodies. There are situations where vaccine antigens associated with limited cross-reactivity to proteins of related microbes would be of priority. In other situations, such as efforts to develop vaccines which offer greater range of protection within a microbial family, for example corona or influenza viruses, vaccines could be manufactured in advance to help control outbreaks caused by emerging species of a particularly pathogenic family. Peptide immunoarrays could be a valuable tool for identification of these cross-reactive antigens to enable the creation of “next generation” vaccines.

### Availability and Implementation

EPIphany was implemented in Python using the Flask webserver framework (http://flask.pocoo.org). EPIphany is available at https://epiphany.usask.ca/epiphany/. The server is “stateless”. Any data uploaded to EPIphany is not retained. Therefore, the user needs to re-upload their data to repeat an analysis, and needs to upload the results from a synoptic analysis for targeted analysis. This policy ensures all data uploaded to EPIphany remains confidential. The source-code for a local-install version can be made available upon request.

## Data Availability

The datasets presented in this article are not readily available because they were used in the current study only to demonstrate the functionality and performance of the software package; they were not used to provide any biological interpretations of the data. Requests to access the datasets should be directed to SN (scott.napper@usask.ca). Please note, a sample dataset is publicly available at https://epiphany.usask.ca/epiphany/download.
