# Supplementary material for: EPIphany—A Platform for Analysis and Visualization of Peptide Immunoarray Data
Source: Front Bioinform. 2021 Jul 7;1:694324. doi: 10.3389/fbinf.2021.694324 (PMC9581008; doi:10.3389/fbinf.2021.694324)
Supplement: Supplementary file 2 [file Image1.pdf]

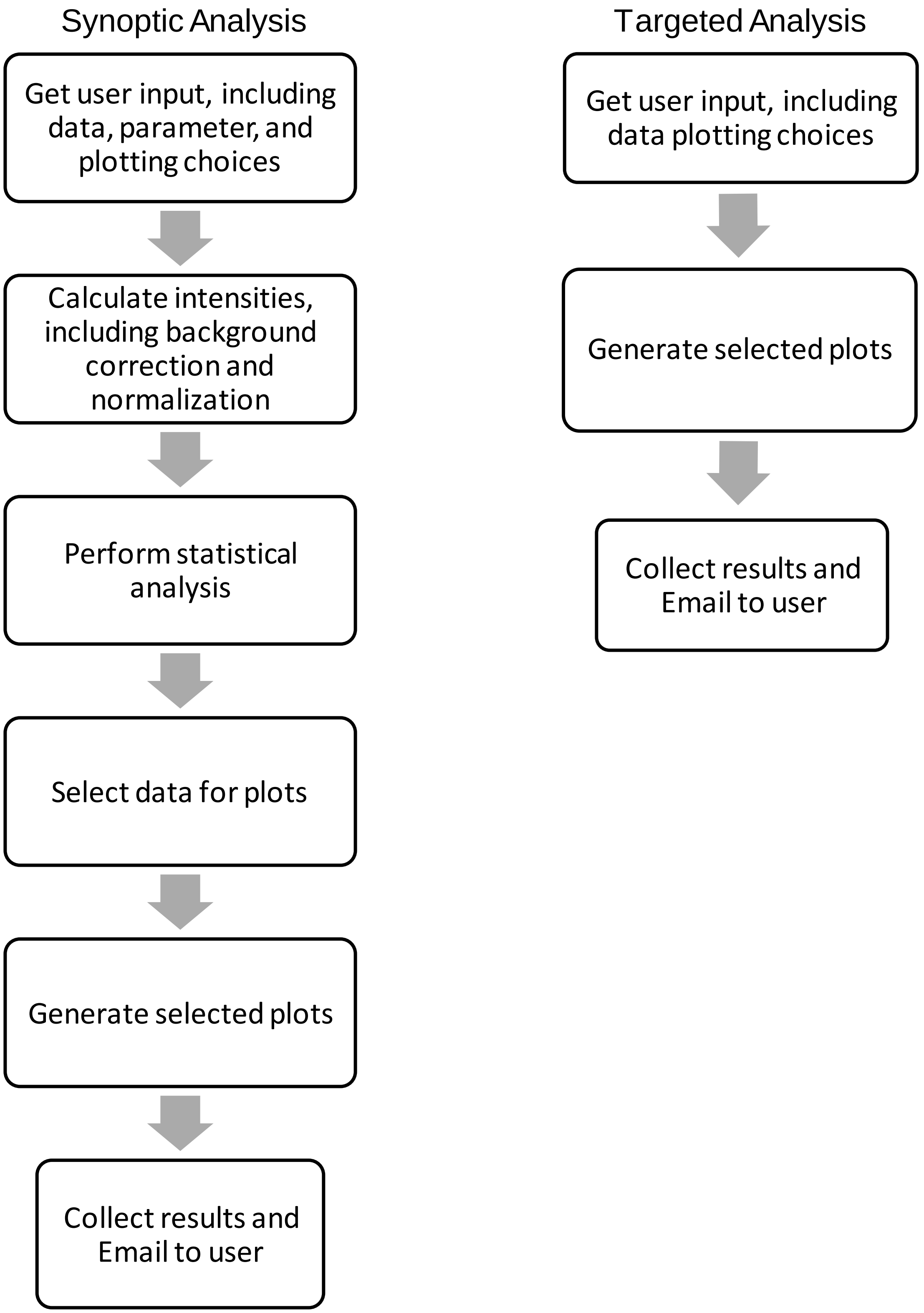

Supplementary Figure 1. Flow diagram depicting the workflow for synoptic and targeted analyses in EPIphany.
